# Characterization of Modified RNA by Top-Down Mass Spectrometry**

**DOI:** 10.1002/anie.201206232

**Published:** 2012-10-08

**Authors:** Monika Taucher, Kathrin Breuker

**Affiliations:** Institut für Organische Chemie and Center for Molecular Biosciences Innsbruck (CMBI), Universität InnsbruckInnrain 80–82, 6020 Innsbruck (Austria)

**Keywords:** collisionally activated dissociation, electron detachment dissociation, mass spectrometry, post-transcriptional modifications, RNA

Ribonucleic acids (RNA) play a vital role in gene expression and frequently carry post-transcriptional modifications (PTMs) that can be important regulators of physiological processes.^1^ For example, a post-transcriptionally modified nucleotide, m^2^A (2-methyladenosine), at position 2503 of the 23S ribosomal RNA was recently shown to play a key role in halting translation.1d More than 100 different naturally occurring modifications are known to date,^2^ but their identification and localization within RNA is generally not possible with high-throughput sequencing methods for the profiling of “transcriptomes”, in which PTM information is lost by reverse transcription of RNA into complementary desoxyribonucleic acid (cDNA).^3^ Methods for the detection of PTMs that do not involve cDNA usually employ enzymatic or chemical hydrolysis, which annihilates sequence information, and often require laborious labeling reactions.3b Herein we present a new, direct approach for the characterization of unknown, modified RNA by top-down mass spectrometry (MS) that immediately reveals the types and sites of all mass-altering modifications without the need for labeling reactions.

Whereas top-down MS of proteins is currently growing into a mature methodology, for which electron capture dissociation (ECD) and collisionally activated dissociation (CAD) of [*M*+*n* H]^*n*+^ ions from electrospray ionization (ESI) provide complementary sequence information,^4^ top-down MS of RNA is far from being established. Chait pointed out that if a sufficient number of fragments are observed, top-down MS can provide a complete description of the primary structure and reveal all mass-altering modifications, as well as any correlations that exist between these modifications.^5^ However, fragmentation techniques for [*M*−*n* H]^*n*−^ ions of RNA, that is, electron detachment dissociation (EDD)^6^ and CAD, are just beginning to be developed.^7^ In previous EDD experiments, we obtained 100 and 97 % sequence coverage from ***d*** and ***w*** fragments (Scheme 1) for 22 and 34 nt sequences, respectively, but only 40 % for 61 nt RNA.7a Moreover, the yields of ***d***, ***w*** fragment ions were generally small compared to those of the oxidized molecular ions from electron detachment. In our proposed EDD mechanism for backbone cleavage into ***d*** and ***w*** fragments, we attribute this low degree of fragmentation to radical stabilization at the nucleobases.7a Furthermore, higher-order RNA gas-phase structure could prevent fragment-ion separation, similar to protein ions withstanding dissociation by ECD.^8^ In “activated ion” ECD,^9^ this limitation is overcome by the use of energetic collisions or infrared (IR) laser radiation to break noncovalent bonds.8a

**Scheme 1:**
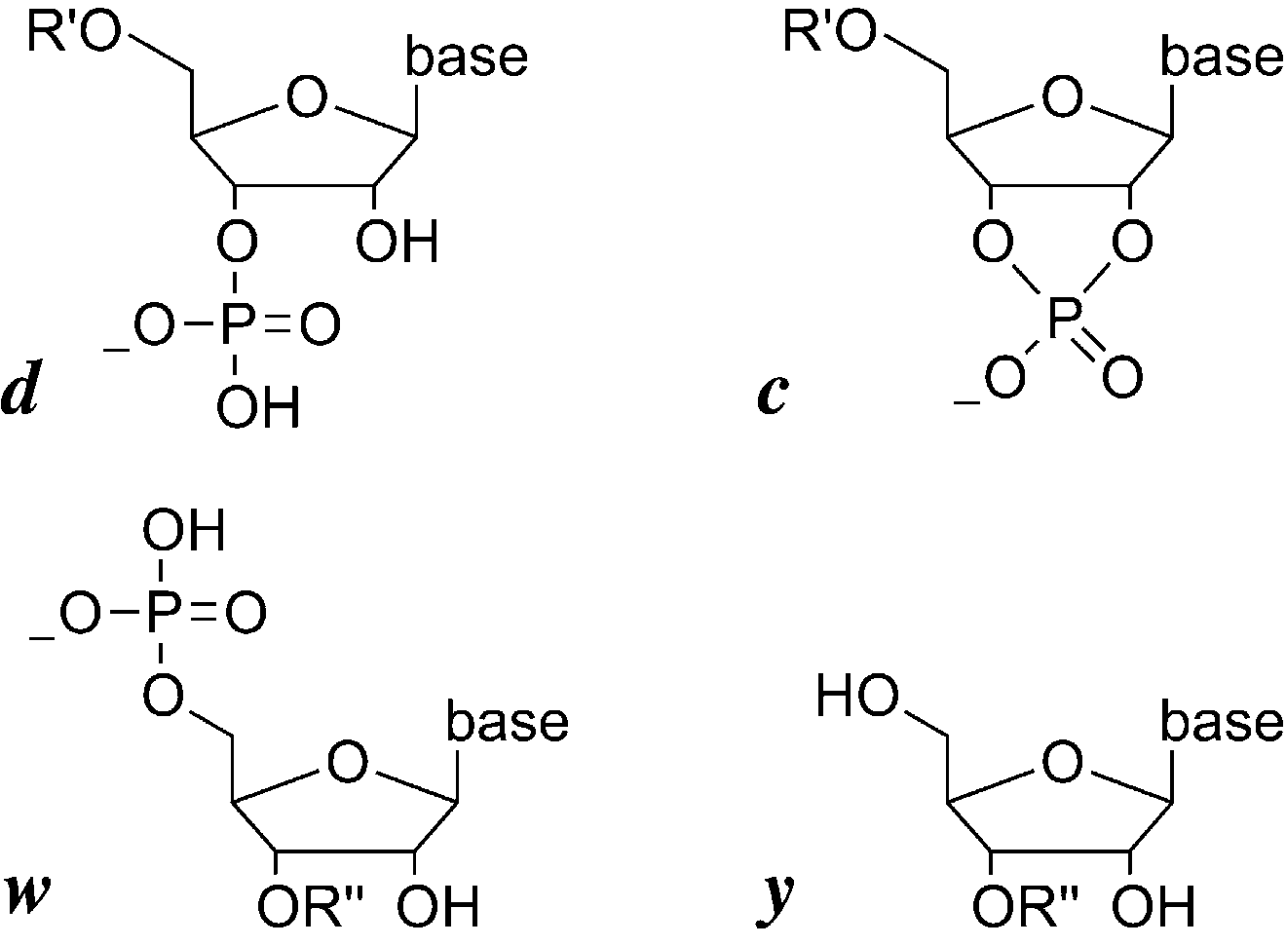
Characteristic fragment-ion types from RNA-backbone cleavage by EDD (***d***, ***w***) and CAD (***c***, ***y***).

Herein we first show that vibrational ion activation, either before or after electron detachment, has no appreciable effect on sequence coverage in EDD of 22 nt RNA, whereas increased negative-ion net charge affords 80 % sequence coverage for highly modified transfer RNA coding for valine (tRNA^Val^, 76 nt) in a single spectrum. We then demonstrate 89 % sequence coverage in a single CAD spectrum of tRNA^Val^ and show how EDD and CAD data can be combined to provide extensive sequence information for the characterization of modified RNA by top-down MS.

Rigorous^10^ collisional activation of 22 nt RNA before electron detachment did not affect sequence coverage (90 % for [*M*−8 H]^8−^ and [*M*−9 H]^9−^, 100 % for [*M*−10 H]^10−^ ions, with and without activation), and gave only marginally higher fragment-ion yields (9, 10, and 14 % of all EDD products, as compared to 9, 11, and 15 % with a laboratory-frame collision energy of 88, 72, and 75 eV for [*M*−8 H]^8−^, [*M*−9 H]^9−^, and [*M*−10 H]^10−^ ions, respectively; Figure 1). The similarity of the spectra with and without collisional activation shows that higher-order gas-phase structure is not a limiting factor in EDD of the 22 nt RNA. The relatively small yield of ***d***, ***w*** fragments at sites other than 15 and 16 for [*M*−8 H]^8−^ and [*M*−9 H]^9−^, and at sites other than 15–19 for [*M*−10 H]^10−^ ions, can instead be attributed to nucleobase ionization energy (IE), as postulated previously7a and supported by calculations.^11^

**Figure 1:**
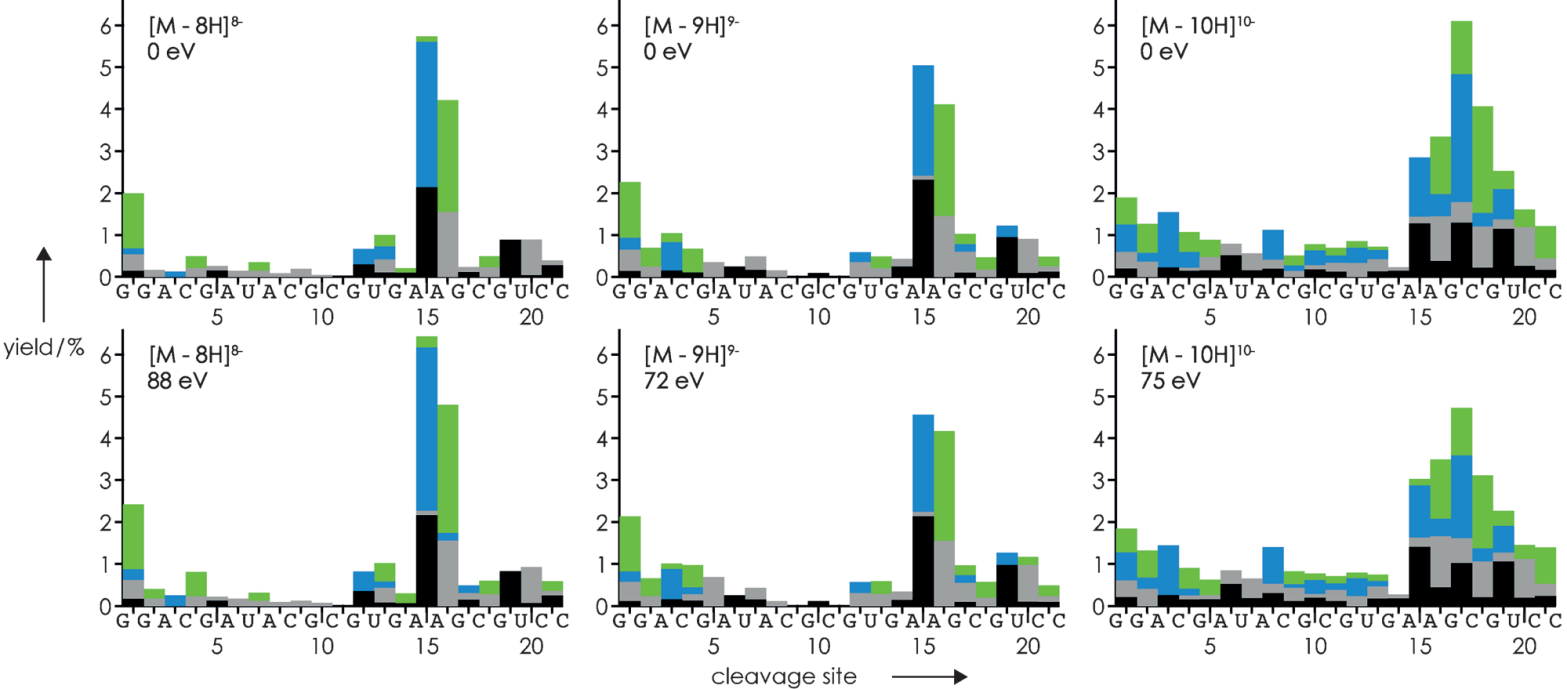
Site-specific yields of ***d***, ***w*** fragments from EDD of [*M*−*n* H]^*n*−^ ions of 22 nt RNA (***d*** in black, ***w*** in gray) and from IR laser activation of [*M*−*n* H]^(*n*−1)−.^ ions formed by electron detachment (***d*** in blue, ***w*** in green), without (top) and with collisional activation prior to EDD (bottom).

Activation by IR laser irradiation (20 % laser power, pulse length: 100 ms) dissociated about 50 % of the radical [*M*−10 H]^9−.^ ions from electron detachment to virtually reproduce the ***d***, ***w*** fragmentation pattern obtained by EDD of the [*M*−10 H]^10−^ ions (Figure 1). No ***c*** or ***y*** ions typically formed by infrared multiphoton dissociation (IRMPD)^12^ or CAD^13^ of [*M*−*n* H]^*n*−^ ions of RNA were observed under these conditions. In contrast, IRMPD of the even-electron [*M*−9 H]^9−^ ions gave only 30 % dissociation at far higher energy (35 %, 200 ms), predominantly into ***c*** and ***y*** ions (Scheme 1) along with some ***a***, ***w***13a but no ***d*** ions, and no dissociation at lower energy (25 %, 50 ms). Similar results were obtained for [*M*−9 H]^9−^ and [*M*−8 H]^8−^ ions (Figure 1). Rather than opening new dissociation channels, vibrational activation of [*M*−*n* H)^(*n*−1)−.^ ions produced the same ***d***, ***w*** ions as EDD of [*M*−*n* H]^*n*−^ ions, consistent with a high-energy transition state towards ***d***, ***w*** ion formation.7a

In our proposed mechanism for RNA-backbone cleavage by EDD,7a the [*M*−*n* H]^(*n*−1)−.^ ions from electron detachment undergo significant structural changes initially driven by charge recombination, including a ribose–nucleobase cyclization reaction that transfers the radical to the nucleobase on the 3′ side of the nucleobase from which the electron is initially detached. Backbone cleavage into noncomplementary, even-electron ***d*** and ***w*** ions then releases an uncharged, radical nucleoside fragment. Intramolecular radical transfer over longer distances, however, does not appear to take place within the [*M*−*n* H]^(*n*−1)−.^ ions, even after vibrational activation, as the ***d***, ***w*** fragmentation patterns from IR laser irradiation of [*M*−*n* H]^(*n*−1)−.^ and EDD of [*M*−*n* H]^*n*−^ ions are not significantly different. Importantly, the data in Figure 1 show that whereas vibrational activation of 22 nt RNA before or after electron detachment had no appreciable effect, increasing the net charge of the precursor ion significantly increased sequence coverage.

An increase in the net charge of an ion leads to an increase in Coulomb repulsion, which should generally lower the energy required for dissociation. For example, the activation energies for covalent-bond dissociation were 1.3 and 0.8 eV for [*M*+H]^+^ and [*M*+2 H]^2+^ ions of bradykinin, respectively,^14^ and those for noncovalent-bond dissociation consistently increased from 1.5 to 3.5 eV when the net charge of a protein complex was decreased from 14+ to 11+.^15^ Such “Coulomb activation” in EDD of RNA should facilitate fragment-ion formation and separation, and produce more random backbone cleavage by leveling differences between nucleobase IEs.7a

“Coulomb activation” requires highly charged precursor ions. We found in this study that the ESI additives quinuclidine, 7-methyl-1,5,7-triazabicyclo[4.4.0]dec-5-ene, 1,8-diazabicyclo[5.4.0]undec-7-ene, and 1,5-diazabicyclo[4.3.0]non-5-ene, all of which gave highly charged ions of 22 nt RNA,^16^ were less effective in producing [*M*−*n* H]^*n*−^ ions of tRNA because of alkali-ion adduction. However, a 1:1 mixture of piperidine, which efficiently suppresses alkali-ion adducts by a mechanism that we have not yet resolved,^16^ and quinuclidine, produced abundant [*M*−*n* H]^*n*−^ ions of tRNA^Val^ with up to 41 net negative charges (0.54 charges/nt; Figure 2 a). This new additive mixture also gave ion yields more than an order of magnitude higher than those observed with triethylamine.7a, 16 We isolated tRNA^Val^ ions with *m*/*z* values between 700 and 920, corresponding to 27–35 net negative charges (Figure 2 b) and subjected them to irradiation with electrons for EDD (Figure 2 c), which gave ***d*** and ***w*** ions from backbone cleavage at 60 out of 75 possible sites (80 % sequence coverage; Figure 3). Collisional activation of the tRNA ions (at laboratory-frame energies of 54–70 eV for ions with 27–35 net charges, respectively) prior to EDD caused about 25 % undesired charged-base loss from the [*M*−*n* H]^*n*−^ ions but did not significantly increase sequence coverage (81 %; see Scheme S1 in the Supporting Information). This result is consistent with that observed for 22 nt RNA (Figure 2) and indicates that higher-order structure is not a limiting factor in EDD of such highly charged tRNA ions. Loss of PTMs from ***d*** or ***w*** ions was not observed in any of the EDD spectra.

**Figure 2:**
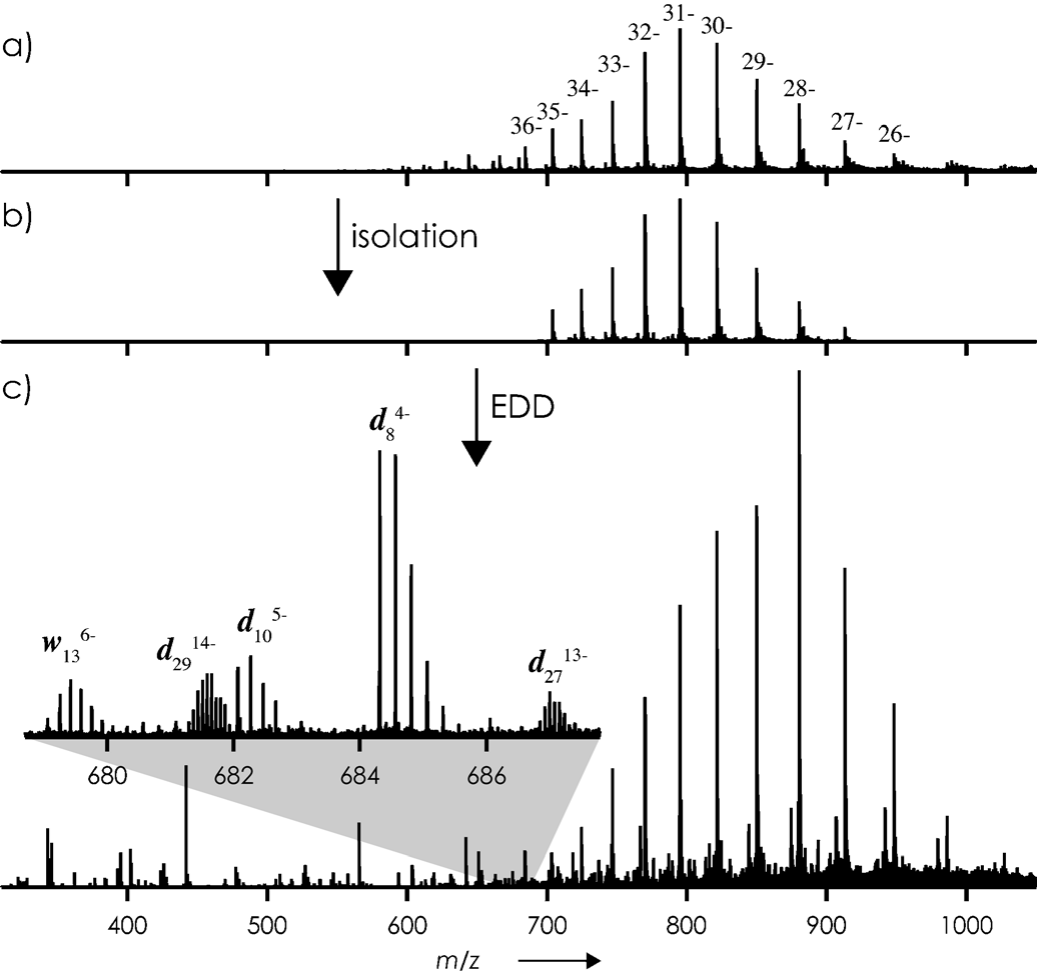
a) ESI mass spectrum of tRNA^Val^ (2 μm) in H_2_O/CH_3_OH (1:1) with piperidine (10 mm) and quinuclidine (10 mm); b) isolation of ions with *m*/*z* values between 700 and 920; c) mass spectrum after exposure of these ions to 28 eV electrons (the inset shows isotopically resolved fragment-ion signals).

**Figure 3:**
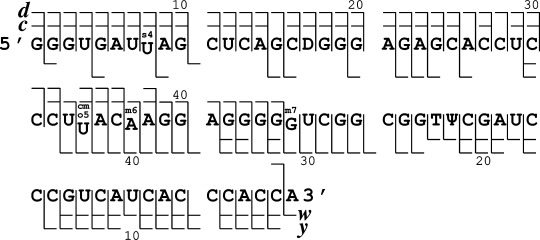
Fragment-ion map illustrating sequence coverage from CAD and EDD of tRNA^Val^. The numbering of ***c***, ***d*** and ***w***, ***y*** fragments starts from the 5′ and 3′ terminus, respectively.

In striking contrast to EDD, CAD of RNA is most informative when the net negative charge of the precursor ions is low.7a,b, 13a The CAD spectrum of [*M*−16 H]^16−^ ions (0.21 charges/nt) of tRNA^Val^ in Figure 4 shows ***c*** and ***y*** ions from backbone cleavage at 67 out of 75 possible sites (89 % sequence coverage; Figure 3); no fragments indicate loss of PTMs. By using [*M*−24 H]^24−^ tRNA ions for CAD in a Paul trap instrument, McLuckey and co-workers recently obtained approximately 60 % sequence coverage.7c

**Figure 4:**
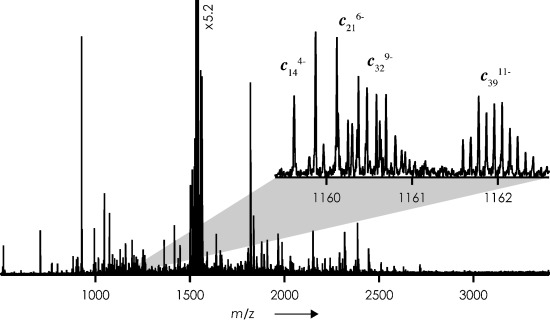
CAD spectrum of [*M*−16 H]^16−^ ions from ESI of tRNA^Val^ (2 μm) in H_2_O/CH_3_OH (1:1) with piperidine (100 mm) and imidazole (100 mm).

Mass values of fragment ions from dissociation of unknown RNA with unknown modifications can in principle be aligned as “mass ladders”^17^ to reveal the exact mass values of all residues and their sequential arrangement (Figure 3). However, proper alignment requires knowledge about whether any given fragment includes either the 3′ or the 5′ terminus; this information is not available from mass values alone. In other words, ***c*** and ***y*** fragments in CAD spectra, and ***d*** and ***w*** fragments in EDD spectra can generally not be distinguished from each other. In this respect, the different fragmentation pathways in CAD and EDD offer a crucial advantage. Whereas CAD of even-electron RNA ions produces complementary ***c*** and ***y*** fragments, the unusual fragmentation of radical RNA ions in EDD gives noncomplementary ***d*** and ***w*** fragments and an uncharged, radical nucleoside fragment.7a Accordingly, the mass difference between ***d*** and ***c*** fragments (which include the original 5′ terminus) is 18.01 Da (H_2_O), and that between ***w*** and ***y*** fragments (which include the original 3′ terminus) is 79.97 Da (HPO_3_). Fragment mass values in the EDD spectrum that are 18.01 and 79.97 Da higher than mass values in the CAD spectrum can thus confidently be assigned as ***d*** and ***w*** ions, respectively (see Table S2 in the Supporting Information). Because this comparison at the same time identifies ***c*** and ***y*** ions in the CAD spectrum, a total of four RNA-fragment “mass ladders” can be generated (Figure 3). Additional confidence in ion assignments comes from a search in the CAD spectrum for fragments whose mass values add up to that of the intact RNA, identifying complementary ***c***, ***y*** ion pairs.7b Likewise, mass values of “quasi-complementary”7a ***d*** and ***w*** ions in the EDD spectrum add up to the mass of the intact RNA plus 97.98 Da (H_3_PO_4_). Implementation of the above search criteria in algorithms for de novo sequencing^18^ should be straightforward.

The combined CAD and EDD data from the two spectra in Figures 2 and 4 provide virtually complete sequence information (99 %) for highly modified tRNA^Val^ (Figure 3). Sample requirements were generally the same as those for top-down MS of proteins using CAD and ECD. Importantly, the data immediately reveal the presence, type, and location of post-transcriptional modifications, although residues of the same mass (e.g., uridine and pseudouridine) cannot be distinguished from each other. However, further RNA characterization can be carried out by using alternative strategies based on mass spectrometry^19^ or chemical reactivity.3b Because our MS approach detects changes in residue mass, it can even identify so far unknown types of PTMs.

In conclusion, we have described herein a straightforward approach for the detailed characterization of highly modified RNA by top-down MS that can readily be implemented in de novo sequencing strategies. In a first step, the exact (to within 1 ppm) mass of an unknown RNA is determined by ESI MS with internal calibration. Next, two separate spectra from RNA dissociation by CAD (of RNA with a low net charge, ca. 0.2 charges/nt) and EDD (of highly charged RNA, ca. 0.5 charges/nt) are recorded and calibrated (see the Supporting Information). Analysis of the two spectra gives two separate lists with monoisotopic fragment mass values. Any values in the EDD mass list that are 18.01 and 79.97 Da higher than values in the CAD mass list are assigned to ***d*** and ***w*** ions, respectively. The corresponding values in the CAD mass list, that is, those that are 18.01 and 79.97 Da lower than values in the EDD mass list, are assigned to ***c*** and ***y*** ions, respectively. Next, values for each ion type (***c***, ***d***, ***w***, ***y***) are separately sorted by mass. This process results in four “fragment mass ladders” that reveal both the exact mass values of RNA residues and their alignment within the sequence. Our new approach can be used to characterize modified as well as unmodified RNA, but should be especially useful for synthetic or post-transcriptionally modified RNA whose analysis by chemical or biochemical methods is rather limited.3b

## Experimental Section

Experiments were performed on a 7 T Fourier transform ion cyclotron resonance mass spectrometer (Bruker, Austria) equipped with an ESI source, a collision cell for CAD, a hollow dispenser cathode for EDD (electron energy: 28 eV), and a CO_2_ laser (10.6 μm, 35 W) for IR activation. Chemicals and tRNA^Val^ (from *Escherichia coli*, GGGUG AU**s^4^U**AG CUCAG C**D**GGG AGAGC ACCUC CCU**cmo^5^U**A C**m^6^A**AGG AGGGG **m^7^G**UCGG CGG**TΨ** CGAUC CCGUC AUCAC CCACC A, modified residues in bold; **s^4^U**: 4-thiouridine, **D**: dihydrouridine, **cmo^5^U**: uridine-5-oxyacetic acid, **m^6^A**: *N*^6^-methyladenosine, **m^7^G**: 7-methylguanosine, **T**: thymidine, **Ψ**: pseudouridine) were purchased from Sigma–Aldrich (Austria). The 22 nt RNA (GGACG AUACG CGUGA AGCGU CC) was prepared by solid-phase synthesis.^20^ For increased statistics, 500 scans were added for each spectrum. For experimental details, see the Supporting Information. SNAP2 (Bruker) and mMass^21^ software were used for data reduction. For Figure 1, the EDD data were normalized to the sum of all EDD products (100 %=[oxidized molecular ions] + ([***d*** ions] + [***w*** ions])/2), and EDD/IR data were normalized to the abundance of the [*M*−n H]^(*n*−1)−.^ ion in the corresponding EDD spectrum.

## Notes

Detailed facts of importance to specialist readers are published as ”Supporting Information”. Such documents are peer-reviewed, but not copy-edited or typeset. They are made available as submitted by the authors.
