# Supplementary material for: Characterization of Modified RNA by Top-Down Mass Spectrometry
Source: Angew Chem Int Ed Engl. 2012 Oct 8;51(45):11289–92. doi: 10.1002/anie.201206232 (PMC3532624; doi:10.1002/anie.201206232)
Supplement: Supplementary file 1 [file anie0051-11289-sd1.pdf]

Angewandte Chemie
Eine Zeitschrift der Gesellschaft Deutscher Chemiker

Supporting Information
© Wiley-VCH 2012
69451 Weinheim, Germany

Characterization of Modified RNA by Top-Down Mass Spectrometry**

*Monika Taucher and Kathrin Breuker**

anie_201206232_sm_miscellaneous_information.pdf

SUPPORTING INFORMATION

Characterization of modified RNA by top-down mass spectrometry

Monika Taucher and Kathrin Breuker*

Desalting of RNA samples

H_2O was purified to 18 MΩ·cm at room temperature using a Milli-Q system (Millipore, Austria), and CH_3OH (Acros, Austria) was HPLC-grade. For desalting, 500 µl of RNA solution (5 to 20 µM) was concentrated to 100 µl using Vivaspin 500 centrifugal concentrators (Sartorius, Germany, PES membrane, MWCO 3000 for 22 nt RNA and MWCO 5000 for tRNA), and 400 µl of an ammonium salt solution (100 mM ammonium acetate in H_2O for 22 nt RNA, 50 mM ammonium citrate in 1:1 H_2O/CH_3OH for tRNA) was added. The process was repeated five times, followed by seven cycles of concentration and dilution with H_2O for 22 nt RNA or 1:1 H_2O/CH_3OH for tRNA. For ESI (flow rate 1.5 µl/min), solutions were diluted to 2 µM RNA in 7:3 H_2O/CH_3OH, with 50 mM piperidine as additive for 22 nt RNA, and 1:1 H_2O/CH_3OH with 10 mM piperidine and quinuclidine each or 100 mM piperidine and imidazole each for tRNA.

tRNA sample consumption

With our instrument configuration, acquisition of the spectra in Figures 2 and 3 each took 1500 seconds (500 scans with 3 seconds/scan), totaling to 75 pmol tRNA consumed for each spectrum. Sample consumption on more modern instruments (nano-ESI source, rf ion transfer, 14 T magnet) can be estimated to be smaller by a factor of at least 200, corresponding to 375 fmol of tRNA.

Calibration of EDD and CAD spectra of tRNA

In treating the $tRNA^{Val}$ (calculated mass 24680.30867 Da for the most abundant isotope) as an unknown, its mass was first determined by FT-ICR MS with internal calibration (standard deviation 0.2 ppm) using $(M - H)^-$ ions of polyethylene glycol 1000 that was added to the ESI solution. An iterative approach was then used for calibration of the EDD spectrum as described below. Using the measured $tRNA^{Val}$ mass (24680.31356 Da for the most abundant isotope), the EDD spectrum was calibrated (standard deviation 0.4 ppm) in the *m/z* range where undissociated $(M - nH)^{n-}$ ions of $tRNA^{Val}$ ions were found. Next, the spectrum was searched for fragment ions that appeared in the spectrum in more than one charge state, of which at least one was within the calibrated *m/z* range. Using the mass values of fragment ions within the calibrated *m/z* range, fragment ion *m/z* values outside this range were calculated and added to the list of $(M - nH)^{n-}$ ion *m/z* values for re-calibration of the EDD spectrum. The parameters from this calibration were also used for external calibration of the CAD spectrum.

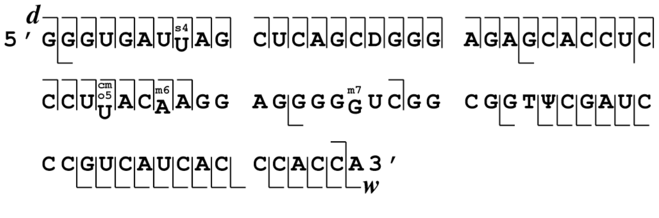

Scheme S1. Fragment ion map illustrating sequence coverage from EDD of $tRNA^{Val}$ with prior collisional activation.

Table S2. Mass spectrometry data from the EDD (left columns, in violet) and CAD (right columns, in blue) spectra in Figures 2 and 3, aligned to highlight characteristic mass differences between ***c*** and ***d*** (18.01 Da) or ***y*** and ***w*** (79.97 Da) fragments (middle column, in black). All mass values refer to the monoisotopic peak.

data from CAD of $tRNA^{Val}$ from E. coli								data from EDD of $tRNA^{Val}$ from E. coli						
yield [a.u.]	accuracy [ppm]	$m_{calculated}$ [Da]	assignment	$m/z_{measured}$	z	$m_{measured}$ [Da]	Δm [Da]	$m_{measured}$ [Da]	z	$m/z_{measured}$	assignment	$m_{calculated}$ [Da]	accuracy [ppm]	yield [a.u.]
								202.0723	1	201.0650	unassigned			36
								206.0559	1	205.0486	unassigned			208
								207.0385	1	206.0312	unassigned			70
								207.0399	1	206.0326	unassigned			181
								208.0463	1	207.0390	unassigned			508
								211.0698	1	210.0625	unassigned			26
								214.0219	1	213.0146	unassigned			34
								218.0673	1	217.0601	unassigned			98
								246.0624	1	245.0551	unassigned			1213
								247.0702	1	246.0629	unassigned			102
								304.0338	1	303.0266	unassigned			44
								306.0252	1	305.0180	unassigned			80
								323.0519	1	322.0446	unassigned			65
								327.0374	1	326.0301	unassigned			38
								329.0525	1	328.0452	unassigned			60
								344.0397	1	343.0325	unassigned			373
								347.0631	1	346.0558	w_1	347.0631	0.09	281
								363.0582	1	362.0509	b_1	363.0580	0.55	96
								385.0078	1	384.0005	unassigned			60
								385.9920	1	384.9847	unassigned			51
								396.0711	1	395.0638	unassigned			226
								403.0185	1	402.0112	unassigned			245
								404.0025	1	402.9952	unassigned			156
								414.0818	1	413.0745	unassigned			46
								425.0142	1	424.0069	c_1	425.0138	0.99	109
								427.0298	1	426.0225	unassigned			157
								443.0247	1	442.0174	d_1	443.0243	0.81	804
								471.0448	1	470.0375	unassigned			54
								510.0443	1	509.0370	unassigned			91
								634.0946	1	633.0874	x_2	634.0938	1.32	57

12	0.20	572.1380	y_2	571.1307	1	572.1379	**79.966**	652.1044	1	651.0971	w_2	652.1044	-0.03	255
								708.0602	1	707.0529	unassigned			73
53	-0.26	770.0612	c_2	769.0541	1	770.0614	**18.010**	788.0718	1	787.0645	d_2	788.0718	0.03	219
							**18.011**	788.0724	2	393.0289			0.75	70
30	0.15	877.1793	y_3	876.1719	1	877.1792	**79.967**	957.1466	2	477.5660	w_3	957.1457	0.96	82
								1014.0856	2	506.0355	unassigned			32
								1034.1367	2	516.0611	unassigned			14
								1037.1120	2	517.5487	unassigned			35
								1037.1592	2	517.5723	unassigned			14
								1038.0970	2	518.0412	unassigned			24
								1053.1065	2	525.5460	unassigned			11
								1053.1550	2	525.5702	unassigned			13
								1054.0932	2	526.0393	unassigned			58
								1069.0916	2	533.5385	unassigned			16
								1077.1182	2	537.5518	unassigned			32
								1078.0953	2	538.0404	unassigned			31
								1093.1126	2	545.5490	unassigned			32
								1094.0996	2	546.0425	unassigned			21
								1117.1257	2	557.5556	unassigned			57
78	0.14	1115.1086	c_3	1114.1012	1	1115.1085	**18.011**	1133.1198	3	376.6993	d_3	1133.1192	0.50	27
							**18.012**	1133.1206	2	565.5530			1.24	274
								1262.1822	2	630.0838	unassigned			20
								1264.1872	2	631.0863	unassigned			71
115	-1.17	1206.2318	y_4	1205.2260	1	1206.2333	**79.966**	1286.1992	3	427.7258	w_4	1286.1982	0.80	31
							**79.966**	1286.1995	2	642.0925			1.03	229
								1299.1915	2	648.5885	unassigned			19
								1302.1918	2	650.0886	unassigned			16
								1321.1896	2	659.5875	unassigned			19
								1341.1610	2	669.5732	unassigned			15
								1343.1370	2	670.5612	unassigned			37
								1353.2178	2	675.6016	unassigned			76
								1383.1475	2	690.5664	unassigned			36
								1421.1353	2	709.5604	unassigned			61
137	-0.19	1421.1339	c_4	709.5598	2	1421.1342	**18.011**	1439.1451	2	718.5653	d_4	1439.1445	0.40	164
							**18.012**	1439.1460	3	478.7081			1.07	46

9	0.79	1511.2731	y_5	754.6287	2	1511.2719	**79.969**	1591.2414	3	529.4065	w_5	1591.2395	1.19	33
55			unassigned	1493.1895	1	1494.1968		1656.2229	2	827.1042	unassigned			106
13			unassigned	823.1048	2	1648.2242		1658.2599	3	551.7460	unassigned			23
								1659.3580	3	552.1121	unassigned			9
								1666.2371	3	554.4051	unassigned			10
								1688.1869	3	561.7217	unassigned			13
67	0.02	1766.1814	c_5	882.0834	2	1766.1813	**18.012**	1784.1935	3	593.7239	d_5	1784.1919	0.89	51
							**18.012**	1784.1936	4	445.0411			0.93	14
15	-0.29	1816.3144	y_6	907.1502	2	1816.3150	**79.967**	1896.2819	3	631.0867	w_6	1897.2880	-0.63	49
14			unassigned	939.0976	2	1880.2097		1963.3006	3	653.4262	unassigned			70
13			unassigned	987.6320	2	1977.2786								
43	-1.71	2095.2339	c_6	1046.6115	2	2095.2375	**18.007**	2113.2445	3	703.4076	d_6	2113.2445	0.04	146
							**18.009**	2113.2465	4	527.3044			0.98	68
14	2.72	2121.3557	y_7	1059.6677	2	2121.3499	**79.973**	2201.3226	2	1099.6540	w_7	2201.3220	0.25	124
							**79.975**	2201.3250	4	549.3240			1.34	11
59	1.29	2401.2592	c_7	1199.6208	2	2401.2561	**18.016**	2419.2716	3	805.4166	d_7	2419.2698	0.76	66
29	-0.06			799.4125	3	2401.2593	**18.012**	2419.2718	4	603.8107			0.83	77
							**18.013**	2419.2720	5	482.8471			0.91	13
35	0.00	2450.4082	y_8	1224.1968	2	2450.4082	**79.930**	2530.3377	2	1264.1616	w_8	2530.3746	-14.56	40
							**79.958**	2530.3664	3	842.4482			-3.23	103
							**79.968**	2530.3767	4	631.5869			0.85	27
							**79.969**	2530.3768	5	505.0681			0.87	8
36	1.89	2723.2616	c_8	1360.6210	2	2723.2565	**18.017**	2741.2736	4	684.3111	d_8	2741.2722	0.50	151
61	0.09			906.7465	3	2723.2614	**18.013**	2741.2740	5	547.2475			0.65	48
35	-0.31	2755.4495	y_9	1376.7179	2	2755.4504	**79.953**	2835.4030	2	1416.6942	w_9	2835.4158	-4.54	26
							**79.967**	2835.4172	4	707.8470			0.47	81
							**79.968**	2835.4180	5	566.0763			0.77	13
11			unassigned	997.8013	3	2996.4256								
62	0.03	3052.3142	c_9	1016.4307	3	3052.3141	**18.012**	3070.3260	4	766.5742	d_9	3070.3247	0.40	263
							**18.012**	3070.3263	5	613.0580			0.50	25
							**18.014**	3070.3279	6	510.7140			1.04	5
32			unassigned	1072.7884	3	3221.3869		3141.4310	2	1569.7082	w_{10}	3141.4411	-3.24	22
								3141.4336	4	784.3511			-2.41	77
								3141.4424	5	627.2812			0.40	16
								3296.3586	6	548.3858	unassigned			9

33	0.34	3390.5273	y_{11}	1129.1681	3	3390.5262	**79.966**	3470.4924	3	1155.8235	w_{11}	3470.4937	-0.36	35
							**79.969**	3470.4948	5	693.0917			0.33	20
							**79.972**	3470.4979	4	866.6172			1.21	52
12	0.02	3397.3616	c_{10}	848.3331	4	3397.3615	**18.012**	3415.3738	5	682.0675	d_{10}	3415.3722	0.48	36
25	0.01			1131.4466	3	3397.3616	**18.014**	3415.3759	6	568.2220			1.11	15
								3774.5144	3	1257.1642	w_{12} (oxidized)			37
30	-0.32	3695.5686	y_{12}	1230.8493	3	3695.5698	**79.962**	3775.5316	6	628.2480	w_{12}	3775.5349	-0.89	10
								3719.4672	4	928.8595	d_{11} (oxidized)			51
13	0.56	3702.4029	c_{11}	924.5929	4	3702.4008	**18.013**	3720.4133	5	743.0754	d_{11}	3720.4135	-0.03	75
23	0.07			1233.1269	3	3702.4026	**18.014**	3720.4166	6	619.0622			0.84	45
27			unassigned	1262.4936	3	3790.5027								
32	-0.49	4001.5939	y_{13}	1332.8580	3	4001.5959	**79.949**	4081.5445	3	1359.5075	w_{13}	4081.5602	-3.87	29
							**79.957**	4081.5532	4	1019.3810			-1.74	86
							**79.965**	4081.5605	6	679.2528			0.06	23
							**79.969**	4081.5646	7	582.0734			1.07	4
								3953.5724	6	657.9215	unassigned			6
29	-0.56	4008.4282	c_{12}	1001.1003	4	4008.4304	**18.014**	4026.4448	7	574.1991	d_{12}	4026.4388	1.51	9
29	-1.07			1335.1369	3	4008.4325	**18.013**	4026.4459	6	670.0670			1.77	27
11	1.91	4313.4695	c_{13}	1077.3580	4	4313.4612	**18.016**	4331.4770	5	865.2881	d_{13}	4331.4800	-0.71	37
65	1.03			1436.8144	3	4313.4650	**18.017**	4331.4824	7	617.7759			0.55	24
							**18.020**	4331.4849	6	720.9069			1.11	53
33			unassigned	1444.1430	3	4335.4507		4425.5851	3	1474.1877	w_{14} (oxidized)			13
34	0.55	4346.6414	y_{14}	1085.6525	4	4346.6390	**79.960**	4426.5993	4	1105.6426	w_{14}	4426.6077	-1.89	62
131	0.27			1447.8728	3	4346.6402	**79.966**	4426.6064	5	884.3140			-0.29	155
							**79.970**	4426.6105	6	736.7611			0.63	96
							**79.976**	4426.6157	7	631.3664			1.82	6
19			unassigned	1115.1408	4	4464.5924								
10			unassigned	897.2867	5	4491.4700								
43			unassigned	900.4855	5	4507.4639								
20	0.95	4562.5557	a_{14}	911.5030	5	4562.5513								
30	0.75			1139.6308	4	4562.5523								
30			unassigned	924.3026	5	4626.5494								
109	1.12	4642.5220	c_{14}	1159.6219	4	4642.5168	**18.008**	4660.5246	7	664.7820	d_{14}	4660.5326	-1.70	10
667	0.82			927.4964	5	4642.5182	**18.020**	4660.5379	8	581.5600			1.15	4
158	1.98	4651.6826	y_{15}	1549.5505	3	4651.6734		5005.5821	7	714.0759	d_{15}	5005.5800	0.43	31

20			unassigned	930.6964	5	4658.5184		5005.5866	8	624.6910			1.32	8
35			unassigned	1001.5593	5	5012.8327		5005.5959	6	833.2587			3.17	33
53	1.46	5261.7652	y_{17}	1752.9119	3	5261.7575	**79.965**	5341.7221	6	889.2797	w_{17}	5341.7315	-1.76	64
							**79.975**	5341.7324	8	666.7093			0.16	20
							**79.976**	5341.7332	7	762.0975			0.31	54
							**79.977**	5341.7343	9	592.5188			0.51	3
41	-0.31	5292.6107	c_{16}	1763.1968	3	5292.6123	**18.010**	5310.6222	7	757.6530	d_{16}	5310.6213	0.17	46
							**18.015**	5310.6270	8	662.8211			1.08	33
								5311.6304	5	1061.3188	unassigned			19
31	3.52	5600.6517	c_{17}	1865.8700	3	5600.6320	**18.026**	5618.6577	6	935.4357	d_{17}	5618.6622	-0.80	83
16	0.47			932.4342	6	5600.6490	**18.013**	5618.6617	7	801.6587			-0.10	97
21	0.32			1119.1227	5	5600.6499	**18.016**	5618.6656	8	701.3259			0.60	52
18	0.18			1399.1554	4	5600.6507	**18.017**	5618.6677	9	623.2891			0.97	13
28			unassigned	1440.9382	4	5767.7818		5647.7440	4	1410.9287	w_{18}	5647.7568	-2.27	12
38	0.42	5896.8430	y_{19}	1964.6062	3	5896.8405	**79.946**	5976.7862	5	1194.3500	w_{19}	5976.8094	-3.88	22
37	0.00			1473.2035	4	5896.8430	**79.949**	5976.7918	4	1493.1907			-2.94	25
							**79.958**	5976.8011	6	995.1262			-1.39	85
							**79.965**	5976.8083	7	852.8225			-0.19	118
							**79.968**	5976.8111	8	746.0941			0.29	127
							**79.978**	5976.8206	9	663.0839			1.88	15
31	2.41	5945.6991	c_{18}	1485.4139	4	5945.6848	**18.008**	5963.6925	5	1191.7312	d_{18}	5963.7097	-2.88	19
21	1.42			1980.8896	3	5945.6907	**18.014**	5963.7046	6	992.9435			-0.86	75
27	0.53			989.9421	6	5945.6960	**18.016**	5963.7125	8	744.4568			0.47	55
							**18.019**	5963.7151	9	661.6277			0.91	14
30			unassigned	1221.5577	5	6112.8250								
9			unassigned	1025.1072	6	6156.6866								
801	0.73	6228.7908	b_{19}	1556.1893	4	6228.7862		6320.8422	5	1263.1612	w_{20} (oxidized)			18
35	3.28	6241.8905	y_{20}	2079.6161	3	6241.8700	**79.983**	6321.8527	6	1052.6348	w_{20}	6321.8568	-0.65	82
22	-0.70			1247.3717	5	6241.8948	**79.965**	6321.8598	7	902.1155			0.47	130
261	-0.91			1559.4668	4	6241.8962	**79.984**	6321.8802	8	789.2278			3.70	39
12			unassigned	1250.3459	5	6256.7660								
31	1.28	6290.7465	c_{19}	1257.1404	5	6290.7385	**18.019**	6308.7580	8	787.5875	d_{19}	6308.7571	0.14	40
18	0.06			897.6707	7	6290.7462	**18.012**	6308.7585	10	629.8686			0.22	5
224	-0.54			1047.4510	6	6290.7499	**18.010**	6308.7597	9	699.9660			0.41	17
9			unassigned	1066.4562	6	6404.7812								

38	-0.46	6546.9318	y_{21}	1635.7264	4	6546.9347	**79.912**	6626.8472	4	1655.7045	w_{21}	6626.8981	-7.68	10
							**79.940**	6626.8743	5	1324.3676			-3.59	23
							**79.947**	6626.8821	6	1103.4731			-2.41	45
17	-0.14	6635.7940	c_{20}	1104.9585	6	6635.7949	**18.011**	6653.8059	7	949.5364	d_{20}	6653.8045	0.21	70
							**18.012**	6653.8065	9	738.3046			0.30	44
							**18.014**	6653.8086	8	830.7188			0.61	88
							**18.015**	6653.8099	10	664.3737			0.80	17
							**18.026**	6653.8211	11	603.8855			2.48	3
							**18.041**	6653.8361	6	1107.9654			4.74	21
8			unassigned	974.6775	7	6829.7936								
9	0.79	6884.8802	a_{21}	1146.4718	6	6884.8747								
30	4.55	6964.8465	c_{21}	1740.1964	4	6964.8148	**18.044**	6982.8590	9	774.8659	d_{21}	6982.8571	0.28	16
24	-0.34			1159.8009	6	6964.8489	**18.012**	6982.8613	10	697.2789			0.61	12
118	-0.52			993.9713	7	6964.8501	**18.014**	6982.8642	7	996.5447			1.02	55
37	0.47	7309.8939	c_{22}	1043.2628	7	7309.8905	**18.000**	7327.8906	11	665.1646	d_{22}	7327.9045	-1.89	6
11	0.17			1217.3082	6	7309.8927	**18.000**	7327.8925	8	914.9793			-1.63	107
							**18.016**	7327.9086	9	813.2048			0.57	86
							**18.020**	7327.9131	10	731.7840			1.18	29
150	0.21	7518.0454	y_{24}	1878.5037	4	7518.0439		6694.9068	6	1114.8105	unassigned			43
77	0.06			1252.0002	6	7518.0450		6932.9210	11	629.2583	w_{22}	6932.9234	-0.35	6
263	0.04			1502.6017	5	7518.0451		6932.9225	10	692.2850			-0.13	12
20	-0.24			1072.9995	7	7518.0472		7252.9671	11	658.3534	w_{23}	7252.9643	0.39	9
23			unassigned	1883.7515	4	7539.0351		7596.9862	6	1265.1571	w_{24} (oxidized)			8
50	0.28	7638.9464	c_{23}	1090.2705	7	7638.9443	**18.012**	7656.9565	12	637.0724	d_{23}	7656.9570	-0.06	3
							**18.014**	7656.9583	11	695.0798			0.17	9
15			unassigned	1298.8365	6	7799.0628								
77	-2.03	7863.0929	y_{25}	1964.7699	4	7863.1088		8001.0081	11	726.3571	d_{24} (oxidized)			11
8	0.43	7983.9939	c_{24}	996.9915	8	7983.9905	**17.984**	8001.9749	10	799.1902	d_{24}	8002.0044	-3.69	27
							**18.023**	8002.0139	12	665.8272			1.18	5
19			unassigned	1616.6020	5	8088.0463								
25	0.75	8168.1280	y_{26}	2041.0247	4	8168.1280								
21	1.13	8289.0352	c_{25}	1035.1210	8	8289.0258	**18.028**	8307.0538	12	691.2472	d_{25}	8307.0457	0.97	9
43	2.42	8513.1816	y_{27}	1701.6249	5	8513.1610		8592.1351	8	1073.0096	w_{27} (oxidized)			11
74	1.87			2127.2842	4	8513.1657		8636.1026	10	862.6030	d_{26}	8636.0982	0.51	58
47	0.67			1215.1607	7	8513.1759		8636.1027	11	784.0930			0.52	29

149	-0.69			1417.8573	6	8513.1875		8636.1053	12	718.6682			0.81	18
73	7.21	8858.2290	y_{28}	2213.5340	4	8858.1651		8636.1278	9	958.5625			3.42	21
26	-1.40			1770.6410	5	8858.2414								
20			unassigned	2219.2816	4	8881.1555								
5	0.91	8923.1290	c_{27}	1114.3828	8	8923.1209	**18.009**	8941.1303	10	893.1058	d_{27}	8941.1395	-1.04	34
							**18.026**	8941.1468	13	686.7732			0.81	10
							**18.028**	8941.1488	11	811.8244			1.04	20
449	-1.41	9163.2703	y_{29}	1526.2066	6	9163.2833		9246.1747	11	839.5541	d_{28}	9246.1808	-0.66	51
57	-3.07			2289.8173	4	9163.2985		9246.1771	14	659.4339			-0.41	3
37	8.02	9469.2956	y_{30}	2366.2976	4	9469.2197		9246.1841	13	710.2377			0.35	18
29	-1.67			1892.8550	5	9469.3114		9548.2304	7	1363.0256	w_{30} (oxidized)			9
								9551.2286	9	1060.2403	d_{29} (oxidized)			22
16	0.27	9534.1956	c_{29}	1058.3475	9	9534.1930	**18.007**	9552.2002	11	867.3746	d_{29}	9552.2061	-0.62	48
							**18.015**	9552.2081	14	681.2933			0.21	18
							**18.017**	9552.2096	13	733.7781			0.37	32
							**18.018**	9552.2114	15	635.8068			0.55	4
261	6.53	9549.2619	w_{30}	2386.2926	4	9549.1996								
135	2.47			1908.8404	5	9549.2384								
98	1.64			1363.1708	7	9549.2463								
37	-0.88			1192.6515	8	9549.2703								
64	-1.43			1590.5387	6	9549.2756								
77			unassigned	2391.7927	4	9571.1999								
22			unassigned	1913.4337	5	9572.2048								
18	-2.11	9828.3587	y_{31}	2456.0876	4	9828.3795								
10	-0.10	9839.2368	c_{30}	1092.2414	9	9839.2378	**18.013**	9857.2512	14	703.0821	d_{30}	9857.2474	0.38	12
							**18.026**	9857.2637	15	656.1436			1.65	4
							**18.029**	9857.2664	13	757.2440			1.93	22
							**18.035**	9857.2732	11	895.1085			2.62	26
								10252.3649	9	1138.1444	w_{32} (oxidized)			12
37	8.36	10173.4061	y_{32}	2542.3230	4	10173.3211	**80.017**	10253.3386	7	1463.7554	w_{32}	10253.3725	-3.31	6
								10162.2893	11	922.8372	d_{31}	10162.2887	0.05	27
								10162.2909	13	780.7074			0.22	41
								10162.3154	12	845.8523			2.63	53
43	1.53	10449.3194	c_{32}	1160.0264	9	10449.3034	**18.032**	10467.3350	13	804.1723	d_{32}	10467.3300	0.48	27
23	0.20			1043.9245	10	10449.3174	**18.020**	10467.3378	15	696.8152			0.74	10

26	5.06	10518.4536	y_{33}	2102.6728	5	10518.4003								
35	4.49			2628.5943	4	10518.4063								
11			unassigned	1061.1216	10	10621.2889								
9	1.29	10675.3784	a_{33}	1185.1443	9	10675.3646								
42	3.95	10755.3447	c_{33}	1791.5431	6	10755.3022								
15	1.56			1343.4087	8	10755.3280								
129	0.03			1074.5272	10	10755.3444								
85	-0.44			1194.0316	9	10755.3495								
183	-2.41			2150.0668	5	10755.3706								
78			unassigned	2154.4643	5	10777.3577								
28			unassigned	2158.0584	5	10795.3282		10942.4422	8	1366.7980	w_{34} (oxidized)			11
64	0.02	10863.5010	y_{34}	2171.6929	5	10863.5007	**79.972**	10943.4731	9	1214.9342	w_{34}	10943.4673	0.53	9
42	-8.67				4	10863.5952	**79.878**							
27			unassigned	2175.6924	5	10883.4985								
25	0.21	11135.3704	c_{34}	1112.5295	10	11135.3680	**18.015**	11153.3835	17	655.0741	d_{34}	11153.3810	0.23	4
							**18.021**	11153.3885	16	696.0795			0.68	10
66	3.36	11208.5484	y_{35}	2240.6949	5	11208.5108	**79.975**	11288.4856	9	1253.2689	w_{35}	11288.5148	-2.58	7
25			unassigned	2244.8936	5	11229.5042		11464.4220	16	715.5191	c_{35}	11464.4229	-0.08	9
								11464.4245	15	763.2877			0.13	31
								11464.4274	14	817.8804			0.39	40
9	0.58	11464.4229	c_{35}	1041.2124	11	11464.4163	**18.019**	11482.4350	14	819.1666	d_{35}	11482.4335	0.13	35
32	0.02			1145.4350	10	11464.4227	**18.019**	11482.4422	16	716.6454			0.76	15
							**18.040**	11482.4628	15	764.4902			2.55	37
57	-1.68	11537.6010	y_{36}	2306.5168	5	11537.6204								
21			unassigned	2310.7121	5	11558.5969								
21	0.89	11769.4642	c_{36}	1175.9381	10	11769.4537	**18.021**	11787.4751	15	784.8244	d_{36}	11787.4748	0.02	13
20	0.89			1068.9431	11	11769.4538	**18.021**							
23	-0.54			1960.5711	6	11769.4705	**18.005**							
63	9.90	11882.6484	y_{37}	2375.4989	5	11882.5307		11960.5934	9	1327.9475	w_{37} (oxidized)			10
24	-0.22			1979.4346	6	11882.6510		11961.6313	11	1086.4138	w_{37} (oxidized)			11
29			unassigned	2379.6973	5	11903.5230								
12	0.80	12112.5324	c_{37}	1210.2450	10	12112.5227								
35	0.21			1100.1318	11	12112.5299								
29	-0.60	12227.6958	y_{38}	2036.9433	6	12227.7032								
119	-3.41			2444.5402	5	12227.7375								

54			unassigned	2448.5375	5	12247.7241								
17			unassigned	2452.5348	5	12267.7102								
7	1.73	12441.5849	c_{38}	1243.1491	10	12441.5633	**17.967**	12459.5301	16	777.7134	d_{38}	12459.5955	-5.25	10
48	0.09			1130.0458	11	12441.5837	**17.946**							
19	4.12	12556.7483	y_{39}	2091.7755	6	12556.6966		12898.8091	16	805.1683	y_{40} (oxidized)			44
53	2.41			2510.3363	5	12556.7180		12898.8101	17	757.7463	y_{40} (oxidized)			18
23			unassigned	2514.7316	5	12578.6945		12898.8182	14	920.3369	y_{40} (oxidized)			38
35	1.30	12786.6323	c_{39}	1161.4123	11	12786.6157		12899.8335	15	858.9816	y_{40}	12899.8165	1.31	49
28	12.69	12899.8165	y_{40}	2578.9233	5	12899.6528		13204.8571	16	824.2963	y_{41}	13204.8578	-0.06	105
46	-2.00			2148.9664	6	12899.8423		13204.8588	17	775.7491			0.08	65
18			unassigned	2152.2960	6	12919.8196		13204.8630	18	732.5962			0.40	25
9	0.36	13131.6798	c_{40}	1093.2990	12	13131.6751		13204.8763	15	879.3178	unassigned			86
19	-1.14			1192.7832	11	13131.6948		14297.9074	12	1190.4850	w_{44} (oxidized)			10
43	4.17	13204.8578	y_{41}	2199.7932	6	13204.8027		15192.9434	14	1084.2030	unassigned			8
19	-1.44			1885.4037	7	13204.8769		15215.0063	14	1085.7789	w_{47}	15215.0515	-2.97	13
23			unassigned	2203.1249	6	13224.7929		18032.5028	23	783.0146	unassigned			19
25	0.22	13460.7323	c_{41}	1222.6954	11	13460.7293		18032.5252	22	818.6530	unassigned			21
14	-0.06			1120.7205	12	13460.7332		18033.4844	25	720.3321	unassigned			6
28	0.88	13533.9103	y_{42}	2254.6425	6	13533.8984		18033.4964	21	857.7306	unassigned			36
60			unassigned	2290.3142	6	13747.9291		18033.5052	24	750.3888	unassigned			14
38			unassigned	1248.8912	11	13748.8834		18378.5533	23	798.0603	unassigned			18
22			unassigned	1373.8824	10	13748.8964		18378.5584	22	834.3817	unassigned			23
28			unassigned	1963.1231	7	13748.9128		19376.6864	24	806.3547	unassigned			18
27			unassigned	2292.8159	6	13762.9389		19679.7092	20	982.9782	unassigned			16
38			unassigned	2295.6448	6	13779.9127		19681.6921	21	936.2162	unassigned			49
10	2.11	13805.7797	c_{42}	1254.0610	11	13805.7506		19681.7086	22	893.6158	unassigned			96
18	0.68			1149.4736	12	13805.7704		19681.7161	25	786.2614	unassigned			31
116	1.78	13913.9360	y_{43}	1986.6943	7	13913.9113		19681.7186	27	727.9453	unassigned			9
175	1.46			2317.9787	6	13913.9156		19681.7307	26	755.9824	unassigned			15
15	0.43			1390.3857	10	13913.9301		19681.7333	23	854.7203	unassigned			59
21	0.04			1263.8959	11	13913.9355		19681.7359	24	819.0651	unassigned			46
19			unassigned	1265.5337	11	13931.9507		19934.7185	23	865.7196	unassigned			16
54			unassigned	1989.6914	7	13934.8910		21351.8261	20	1066.5840	w_{66}	21351.8855	-2.78	8
101			unassigned	2321.4761	6	13934.9004		21351.8494	21	1015.7475			-1.69	19
13			unassigned	1991.9724	7	13950.8575		21351.8914	23	927.3358			0.28	19

36			unassigned	2324.6348	6	13953.8524		21351.8991	22	969.5336	unassigned			20
21	-0.63	14150.8272	c_{43}	1178.2291	12	14150.8361		22143.6969	24	921.6468	unassigned			12
27	9.90	14219.9613	y_{44}	2368.9628	6	14219.8206		24319.2521	26	934.3486	unassigned			10
62	0.78			2030.4142	7	14219.9502		24324.2271	24	1012.5022	w_{75}	24324.2333	-0.25	15
16	-1.37			1776.4903	8	14219.9808		24324.2369	25	971.9622			0.15	23
17			unassigned	2372.4583	6	14240.7935		24401.1920	30	812.3658	unassigned			12
30			unassigned	2033.4155	7	14240.9597		24403.2126	29	840.4828	unassigned			14
20	-0.49	14495.8746	c_{44}	1206.9829	12	14495.8817		24420.2122	30	812.9998	d_{75}	24420.1946	0.72	30
11	0.11	14840.9220	c_{45}	1235.7361	12	14840.9204		24420.2286	29	841.0696			1.40	18
14			unassigned	1245.2379	12	14954.9427		24420.2465	31	786.7426			2.13	8
23	-3.70	15135.0852	y_{47}	2161.1558	7	15135.1413		24456.1772	23	1062.3048	unassigned			6
39			unassigned	1260.4947	12	15138.0239		24458.2471	25	977.3226	unassigned			22
25	-1.90	16380.2456	y_{51}	2046.5273	8	16380.2766		24499.2424	24	1019.7945	unassigned			39
52	-2.01	17030.3343	y_{53}	1891.2559	9	17030.3686		24501.2071	26	941.3468	unassigned			158
25			unassigned	1893.6996	9	17052.3621		24503.1603	23	1064.3475	unassigned			10
27	3.52	17359.3868	y_{54}	1927.8067	9	17359.3257		24503.2054	25	979.1209	unassigned			88
14			unassigned	1947.8092	9	17539.3479		24503.2254	33	741.5147	unassigned			15
11			unassigned	1949.8109	9	17557.3632		24503.2267	30	815.7669	unassigned			72
14			unassigned	1951.3691	9	17571.3872		24503.2284	28	874.1080	unassigned			144
29	-1.46	17704.4342	y_{55}	1769.4387	10	17704.4601		24503.2289	29	843.9317	unassigned			109
118	-2.18			1966.1564	9	17704.4729		24503.2564	27	906.5207	unassigned			109
68			unassigned	1968.4891	9	17725.4678		24504.2236	32	764.7497	unassigned			23
27			unassigned	1970.7057	9	17745.4168		24504.2368	31	789.4520	unassigned			41
18	1.22	18378.5342	y_{57}	1669.7665	11	18378.5119		24504.2403	34	719.7057	unassigned			6
152	0.49			1836.8452	10	18378.5251		24522.8149	32	765.3307	unassigned			10
23	-2.35			2041.0569	9	18378.5774		24634.2821	32	768.8140	unassigned			13
70			unassigned	1839.0436	10	18400.5084		24634.2971	33	745.4866	unassigned			7
30			unassigned	1840.8381	10	18418.4542		24634.3095	31	793.6479	unassigned			19
10			unassigned	1489.8871	13	19381.6270		24650.3129	33	745.9719	unassigned			8
99			unassigned	1500.4281	13	19518.6605		24650.3214	28	879.3613	unassigned			102
44			unassigned	1501.7340	13	19535.6360		24650.3550	27	911.9688	unassigned			33
240	1.92	19681.7113	y_{61}	1512.9676	13	19681.6735		24651.3269	30	820.7036	unassigned			52
154			unassigned	1514.5067	13	19701.6814		24651.3416	29	849.0390	unassigned			57
21			unassigned	1795.3163	11	19759.5593		24652.2919	32	769.3768	unassigned			21
20			unassigned	1797.2292	11	19780.6008		24652.3111	31	794.2286	unassigned			43

51			unassigned	1804.6872	11	19862.6389		24667.2796	23	1071.4831	M (oxidized)			10
54			unassigned	1806.0532	11	19877.6648		24665.2830	24	1026.7129	M (oxidized)			46
63			unassigned	1807.5090	11	19893.6794		24669.2563	25	985.7630	M	24669.2807	-0.99	127
20			unassigned	1808.2360	11	19901.6757		24669.2513	26	947.8101	M	24669.2807	-1.19	338
54			unassigned	1809.6003	11	19916.6835		24668.2861	27	912.6330	M (oxidized)			599
10			unassigned	1810.5887	11	19927.5561		24669.2904	28	880.0388	M	24669.2807	0.39	902
25			unassigned	1811.2354	11	19934.6690		24669.3121	29	849.6587	M	24669.2807	1.27	664
29			unassigned	1818.1554	11	20010.7900		24669.2999	30	821.3027	M	24669.2807	0.78	594
37	0.62	20026.7587	y_{62}	1667.8883	12	20026.7463		24669.2876	31	794.7762	M	24669.2807	0.28	461
529	-0.42			1819.6079	11	20026.7672		24669.2848	32	769.9079	M	24669.2807	0.16	294
297			unassigned	1821.6085	11	20048.7732		24669.2829	33	746.5468	M	24669.2807	0.09	176
24			unassigned	1822.6129	11	20059.8214		24669.2872	34	724.5600	M	24669.2807	0.26	88
118			unassigned	1823.4254	11	20068.7597		24669.2825	35	703.8294	M	24669.2807	0.07	33
19			unassigned	1824.6051	11	20081.7363								
46			unassigned	1825.3339	11	20089.7527								
19			unassigned	1835.8926	11	20205.8990								
45			unassigned	1853.3564	11	20398.0003								
27			unassigned	1855.1728	11	20417.9806								
13			unassigned	1488.7005	14	20855.9092								
16	-0.37	21946.0191	y_{68}	1687.1487	13	21946.0272								
52	1.14	23248.1468	y_{72}	1659.5727	14	23248.1202								
32			unassigned	1661.2177	14	23271.1503								
127			unassigned	1521.2527	16	24356.1597								
162			unassigned	1522.2536	16	24372.1736								
162			unassigned	1523.5674	16	24393.1945								
231			unassigned	1524.3180	16	24405.2040								
224			unassigned	1525.4426	16	24423.1982								
138			unassigned	1526.6915	16	24443.1811								
129			unassigned	1528.5099	16	24472.2748								
136			unassigned	1529.5728	16	24489.2815								
824			unassigned	1530.6320	16	24506.2289								
163			unassigned	1531.2585	16	24516.2521								
592			unassigned	1531.6952	16	24523.2399								
704			unassigned	1532.5696	16	24537.2306								
530			unassigned	1533.9444	16	24559.2274								

8			unassigned	1637.2846	15	24574.3788								
284			unassigned	1535.1934	16	24579.2101								
80			unassigned	1638.4818	15	24592.3356								
38			unassigned	1639.8153	15	24612.3382								
203			unassigned	1538.8238	16	24637.2966								
374			unassigned	1539.8871	16	24654.3095								
3015	1.98	24669.2807	M	1540.8197	16	24669.2318								
